# Spatial-Temporal Footprints Assessment and Driving Mechanism of China Household Diet Based on CHNS

**DOI:** 10.3390/foods10081858

**Published:** 2021-08-11

**Authors:** Yanling Long, Runzhi Hu, Tuo Yin, Pengxiang Wang, Jiamin Liu, Tahir Muhammad, Xiuzhi Chen, Yunkai Li

**Affiliations:** 1 College of Water Resources and Civil Engineering, China Agricultural University, Beijing 100083, China; lylforoffice@gmail.com (Y.L.); hurunzhi@cau.edu.cn (R.H.); tuoyin@cau.edu.cn (T.Y.); w1208742578@gmail.com (P.W.); liujiamin@cau.edu.cn (J.L.); engineerkakar95@cau.edu.cn (T.M.); yunkai@cau.edu.cn (Y.L.); 2 Engineering Research Center for Agricultural Water-Saving and Water Resources, Ministry of Education, Beijing 100083, China

**Keywords:** footprint, food consumption, China, CHNS, household

## Abstract

Food consumption is closely associated with resource consumption and environmental sustainability. An unreasonable dietary pattern would cause great pressure or damage to resources and the environment. It is particularly important to reduce the negative impact of household food consumption on resources and the environment while simultaneously ensuring people’s nutrient intake and health. This study applied the China Health and Nutrition Survey (CHNS) database to quantitatively study the spatial-temporal analysis of multiple footprints of household food consumption at multiple scales and explored the driving mechanism of the multiple footprints. The results showed that, except land footprint (LF), the other four types of footprints all decreased at varying degrees; the water footprint (WF), carbon footprint (CF), nitrogen footprint (NF) and energy footprint (EF) decreased by 18.24%, 17.82%, 12.03% and 20.36%, respectively, from 2000 to 2011; multiple footprints of food consumption of household in Guizhou was the highest among the 12 provinces involved in the study; this shows that resource consumption (water, energy and land resource) and environmental influences (CO_2_ emissions and nitrogen emissions) brought by food consumption of per household in Guizhou are much greater than in other provinces, which has a negative influence on sustainable development; by analyzing the driving factors of multiple footprints, it is shown that nutrient intake, household attributes, educational level and health conditions were significantly correlated to multiple footprints. Among them, nutrient intake has greater impact on the multiple footprints of Chinese household food consumption. By comparing multiple footprints of different dietary patterns, it was found that the current Chinese dietary pattern would cause excessive resource consumption, which would bring more pressure on resources and the environment. Adjusting household living habits would possibly reverse the unsustainable situation, such as reducing the consumption of animal-derived foods and adjusting the dietary pattern of households with a higher educational level and income status. Chinese Dietary Guidelines 2016 has better sustainability; the promotion of this dietary pattern across the country would help China to relieve the pressure on resources and environment from the consumer side, promoting the realization of sustainable development.

## 1. Introduction

Food consumption would have a profound impact on human health, resource consumption and environment sustainability [1,2]. With the rapidly growing population and higher living standards, food production due to increased demand would face greater pressure [3]. Agricultural production directly pressures water, energy, and land resources [4,5,6], and causes environmental issues including threats to biodiversity; increase in GHG emissions; and harm to marine, freshwater and terrestrial ecosystems [7,8,9]. Thus, although complicated, there is an urgent requirement to fix these issues, guaranteeing water, energy and food security to achieve sustainable development while satisfying nutrition requirements.

Environmental footprints have been used widely to evaluate resources and environmental performance [10]. In order to quantify the impacts of food consumption on resources and environment, this study considered five footprint indicators, including water footprint (WF), carbon footprint (CF), nitrogen footprint (NF), energy footprint (EF) and land footprint (LF). WF is defined as the volume of water needed for the production of goods and services consumed by inhabitants [11]. CF stands for a certain amount of direct as well as indirect CO_2_ emissions relevant to climate change and is associated with human production or consumption activities [12]. NF is used to quantify nitrogen emissions during production processed by calculating the potential loss of radioactive nitrogen [13]. EF represents the primary energy consumption and is used to calculate the energy embodied in goods or services [14,15]. Finally, LF is the amount of land used to produce goods and services [16,17].

Numerous studies of footprint had put forward effective evaluation methods of resource consumption and environmental impacts, but no study has been carried out considering a spatial-temporal analysis of multiple footprints at multiple scales of food consumption. 

Some studies analyzed footprints in certain national or regional scales. Masud et al. [18] assessed the WF of barley of Alberta in western Canada. Zhai et al. [19] calculated EF and WF of plant-foods in China and analyzed its environmental impact on the North China Plain and the Northeast Plain. A simulation and analysis model of China’s energy consumption was established by Li [20] for the first time, which was used to calculate the flow efficiency of China’s agricultural energy consumption. Ruiter et al. [17] calculated the agricultural LF of the United Kingdom from 1986 to 2011. Liu et al. [21] compared the impact of different food consumption patterns on China’s water demand. A geospatial approach was developed by Jin et al. [22] for estimating the EF, and it was tested for crops in Delaware. Moreover, Naja et al. [23] evaluated and compared the environmental footprints of food consumption patterns among Lebanese adults.

Some studies considered individual or multiple footprints; for instance, Eduardo et al. [24] calculated the LF of rice and maize food loss and waste in Brazil. Xue et al. [25] compared the CF and NF of eight food types. Kashyap et al. [26] analyzed the variability in CF among the five agro-climatic zones and farm sizes of Punjab, India. A common framework was developed by Oita et al. [27] for the purpose of making comparisons to examine the food NF and phosphorus footprint of China, India, and Japan from 1961 to 2013. Wang et al. [28] evaluated the effects of 11 kinds of foods and 16 adjusted dietary scenarios on obesity and CF. Vanham et al. [29] classified various European diets into current diet, healthy diet, vegetarian diet and omnivorous diet to evaluate the WF of each dietary pattern. Esteve-Llorens et al. [30] quantified the CF of the Atlantic diet. Blas et al. [31] investigated and compared the nutritional and water implications of the current food consumption of Spanish households with the recommended Mediterranean diet. Kovacs et al. [32] modeled the CF of the dietary guidelines from seven different countries. Thus, it has become necessary to explore the driving factors and mechanisms of dietary resource consumption more comprehensively and systematically.

The objectives of this study were to: (I) analyze the multiple footprints of food consumption at household scale and explore its spatial-temporal analysis at multiple scales; (II) determine the driving factors of multiple footprints of household food consumption; (III) find a resource-sustainable diet which could guarantee the household demand of nutrient intake, reduce resource consumption and lower greenhouse gas emissions simultaneously. 

## 2. Materials and Methods

### 2.1. Research Area

**Figure 1:**
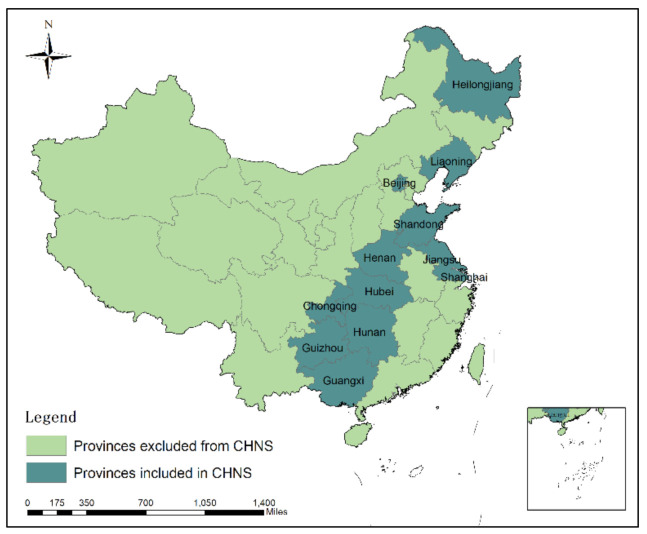
Geographical distribution of study regions.

China is the world’s largest developing country and the world’s second largest economy (China’s GDP was USD 14.28 trillion in 2019, ranked 2nd in the world). China is also the most populated country in the world (1.4 billion in 2019) [22]. This study involved 12 provinces of China (Figure 1), which included Beijing, Liaoning, Heilongjiang, Shanghai, Jiangsu, Shandong, Henan, Hubei, Hunan, Guangxi Zhuang Autonomous Region, Chongqing and Guizhou as research areas. The population of these 12 provinces accounts for over 40% of the China’s total population. The research included all the food consumption survey data that can be collected from CHNS, of 63,550 households, and the last five years provided by the database (2000, 2004, 2006, 2009, and 2011).

### 2.2. Food Classification

As is shown in Table 1, based on the statistics of the CHNS database, fifteen kinds of foods were divided into two categories: plant-based foods and animal-derived foods. Through elaboration of two-level food categories, among them, the primary classification included cereals and starchy foods; legumes and its products; vegetables and fruit; animal-derived foods. In Chinese households, these are the major types of food consumed [21].

### 2.3. Methods

In order to quantify the footprint of the whole process of food consumption more accurately, this paper adopts the life cycle assessment method in the footprint assessment to calculate the footprints of plant-based and animal-derived foods at all stages of the process, from production to consumption, and establish a calculation model of multiple footprints [33]. The specific formulae are as follows:

(1) Dietary WF:

(1) $$\mathrm{DWF}=\sum_{i=1}^{n}P_{i}{\times \mathrm{WF}}_{i}$$

where $P_{i}$ represents the consumption of product *i*, g/cap/d, ${\mathrm{WF}}_{i}$ represents the WF per unit yield of product *i*, $\mathrm{and}\;m^{3}/\mathrm{kg}$, DWF represents the WF, $m^{3}/\mathrm{cap}/d$ [34].

(2) Dietary CF:

(2) $$\mathrm{DCF}=\sum_{i=1}^{n}P_{i}{\times \mathrm{CF}}_{i}$$

where $P_{i}$ represents the consumption of product *i*, g/cap/d, ${\mathrm{CF}}_{i}$ represents the CF per unit yield of product *i*, $\mathrm{and}\;{\mathrm{kgCO}}_{2}\mathrm{eq}/\mathrm{kg}$, DCF represents the WF, ${\mathrm{kgCO}}_{2}\mathrm{eq}/\mathrm{cap}/d$.

(3) Dietary NF:

The NF includes nitrogen emissions in the whole life cycle of the food from production to processing, which can be divided into the NF of food production and food consumption [35].

(3) $${\mathrm{NF}}_{\mathrm{food}}=\sum_{i=1}^{n}{\mathrm{NF}}_{\mathrm{food}\;i}=\sum_{i=1}^{n}\left({\mathrm{NF}}_{\mathrm{consumption}\;i}{+\mathrm{NF}}_{\mathrm{production}\;i}\right)$$

(4) $${\mathrm{NF}}_{\mathrm{consumption}\;i}{=S}_{\mathrm{protein}\;i}{\times \mathrm{NC}}_{\mathrm{protein}\;i}{-W}_{\mathrm{food}\;i}$$

where $S_{\mathrm{protein}\;i}$ represents the amount of protein supplied by food in t; ${\mathrm{NC}}_{\mathrm{protein}\;i}$ is the nitrogen content of protein, which by default is 16%; $W_{\mathrm{food}}$ represents the loss and waste of consumption in ton in the process; i represents different types of food.

(5) $${\mathrm{NF}}_{\mathrm{production}\;i}{=\mathrm{NF}}_{\mathrm{consumption}\;i}{\times \mathrm{VNF}}_{\mathrm{food}\;i}$$

${\mathrm{VNF}}_{\mathrm{food}\;i}$ refers to the virtual nitrogen content of different foods and the virtual nitrogen content discharged into the environment during the process from production to consumption, which mainly exists in the volatilization of chemical fertilizers, runoff, crop harvest loss, processing loss, loss of animal manure and urine, etc. [36].

(4) Dietary EF:

(6) $$\mathrm{DEF}=\sum_{i=1}^{n}P_{i}{\times \mathrm{EF}}_{i}$$

where DEF represents the dietary EF, MJ/cap/d, $P_{i}$ represents the consumption of product *i*, g/cap/d, ${\mathrm{EF}}_{i}$ represents the EF per unit yield of product *i*, MJ/kg.

(5) Dietary LF:

(7) $$\mathrm{DLF}=\sum_{i=1}^{n}P_{i}{\times \mathrm{LF}}_{i}$$

where DLF represents the dietary EF, ${\mathrm{ghm}}^{2}/\mathrm{cap}/y$, $P_{i}$ represents the consumption of product *i*, g/cap/d, ${\mathrm{LF}}_{i}$ represents the LF per unit yield of product *i*, ${\mathrm{ghm}}^{2}/t$. 

Footprint intensity of nutrient intake is calculated as follows:

(8) $${\mathrm{FI}}_{\mathrm{nutrition}}=\frac{F}{\sum_{i=1}^{n}P_{i}{\;\times \;\mathrm{NC}}_{i}}$$

where $FI_{nutrition}$ represents multiple footprint (including WF, CF, NF, EF and LF) intensity of nutrient intake (including energy, protein, insoluble dietary fiber and cholesterol). There are 20 kinds of footprint intensity for nutrient intake in the paper (including ${\mathrm{WFI}}_{e}$, ${\mathrm{WFI}}_{p}$, ${\mathrm{WFI}}_{f}$, ${\mathrm{WFI}}_{c}$, ${\mathrm{CFI}}_{e}$, ${\mathrm{CFI}}_{p}$, ${\mathrm{CFI}}_{f}$, ${\mathrm{CFI}}_{c}$, ${\mathrm{NFI}}_{e}$, ${\mathrm{NFI}}_{p}$, ${\mathrm{NFI}}_{f}$, ${\mathrm{NFI}}_{c}$, ${\mathrm{EFI}}_{e}$, ${\mathrm{EFI}}_{p}$, ${\mathrm{EFI}}_{f}$, ${\mathrm{EFI}}_{c}$, ${\mathrm{LFI}}_{e}$, ${\mathrm{LFI}}_{p}$, ${\mathrm{LFI}}_{f}$, ${\mathrm{LFI}}_{c}$). F represents dietary footprint; $P_{i}$ refers to the consumption of product i at different scales, kg/cap/d; ${\mathrm{NC}}_{i}$ represents the nutrition content of the edible part of the per-unit weight of food for energy, protein, insoluble dietary fiber and cholesterol, MJ/kg, g/kg, g/kg, and mg/kg.

### 2.4. Driver Factor Screening and Analysis

To analyze the factors for the differences between dietary WF, CF, NF, EF and LF at a household scale in China, and to compare the influence degree of these factors, 65 independent variables of 6 categories were selected from the CHNS database (as shown in Table 2). They are household attributes (V1 to V12), nutritional intake ratio (V13 to V16), labor and income status (V17 to V29), health and medical conditions (V30 to V49), educational level and social life (V50 to V54), and living habits (V55 to V65). The 65 proposed independent variables which were screened out and a correlation analysis was performed with multiple footprints and footprint intensities of nutrient intake at a household scale. The Spearman coefficient was calculated, and the significant variables were screened by significance test.

### 2.5. Data Sources and Processing

**Figure 2:**
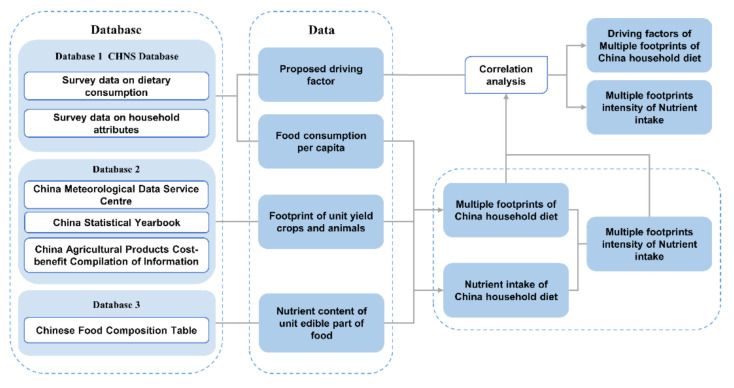
Research methods and processes.

As is shown in Figure 2, the study was based on the food consumption survey data of 63,550 household in 12 provinces of China during 2000, 2004, 2006, 2009 and 2011 from CHNS [37]. The food yield per unit area footprint value at the national and provincial scales was obtained by Compilation of Cost–Benefit Data of National Agricultural Products of China [38]. The multiple footprints of household food consumption were calculated. The nutrition content of the edible part of food unit was obtained from the Chinese Food Composition Table [39,40] of the Institute of Nutrition and Health, Chinese Center for Disease Control and Prevention, and four nutrients including energy, protein, cholesterol and insoluble dietary fiber were selected to calculate the intensity of nutrient footprint in household food consumption. The independent variables of dietary footprint, nutrients footprint intensity and driving factors were imported into SPSS 25.0 for correlation analysis.

## 3. Results

### 3.1. Spatial-Temporal Analysis for Multiple Footprints of Food Consumption

**Figure 3:**
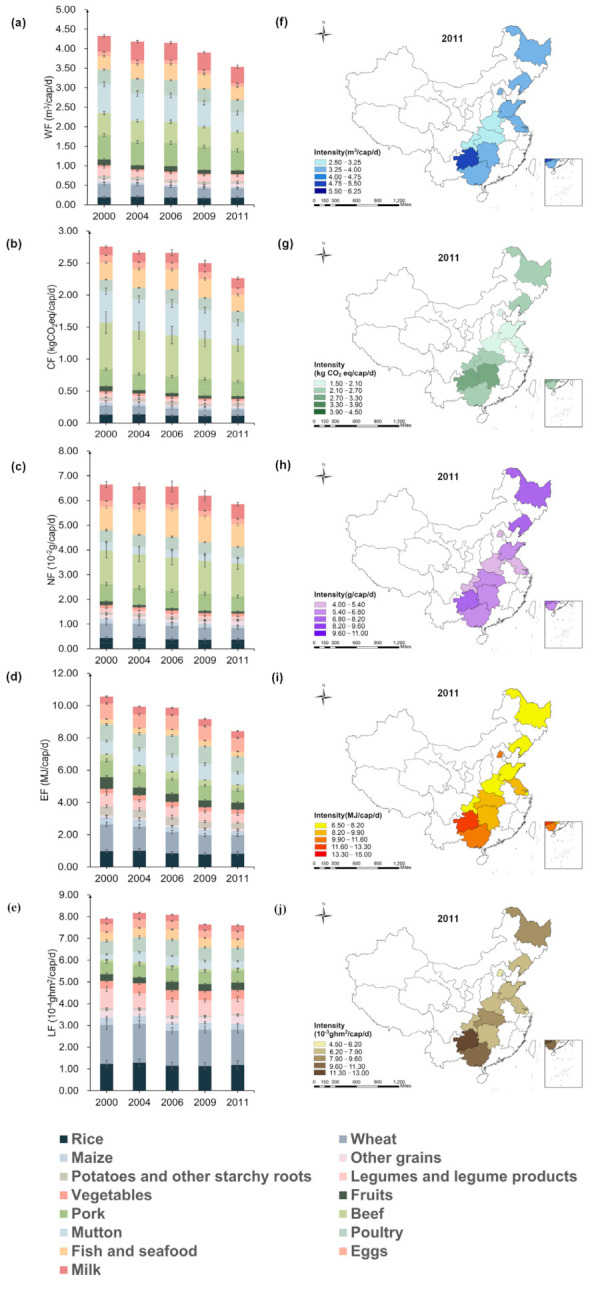
Spatial-temporal characteristics of dietary footprints of Chinese household ((**a**,**f**): WF; (**b**,**g**): CF; (**c**,**h**): NF; (**d**,**i**): EF; (**e**,**j**): LF; The error lines in the figure are standard errors).

As shown in Figure 3, the dietary WF of China’s household decreased from 4.33 $m^{3}/\mathrm{cap}/d$ to 3.54$\;m^{3}/\mathrm{cap}/d$, by −18.24%. Dietary CF decreased from 2.75$\;{\mathrm{kg}\;\mathrm{CO}}_{2}\mathrm{eq}/\mathrm{cap}/d$ to 2.26 ${\mathrm{kgCO}}_{2}\mathrm{eq}/\mathrm{cap}/d$, by −17.82%, the maximum value for both of these factors were obtained in 2000. In addition, the NF decreased from 6.65$\;{\times \;10}^{-2}g/\mathrm{cap}/d$ to 5.85$\;{\times \;10}^{-2}g/\mathrm{cap}/d$, by −12.03%. The EF decreased from 10.56 MJ/cap/d to 8.41 MJ/cap/d, by −20.36%. The LF showed the increasing trend first and then decreased, decreasing from 7.91$\;{\times \;10}^{-4}\;{\mathrm{ghm}}^{2}/\mathrm{cap}/d$ to 7.60 ${\times \;10}^{-4}\;{\mathrm{ghm}}^{2}/\mathrm{cap}/d$, which reached the maximum value of 8.18 ${\times \;10}^{-4}\;{\mathrm{ghm}}^{2}/\mathrm{cap}/d$ in 2004. However, the change was not much obvious. Except LF, footprints produced by the food consumption of animal-derived foods were mostly higher than plant-based foods. During 2000–2011, the proportion of WF in plant-based foods decreased from 26.87% to 24.96%, the proportion of CF in plant-based foods decreased from 20.91% to 18.64%, the proportion of NF in plant-based foods decreased from 28.89% to 25.86%, the proportion of EF in plant-based foods decreased from 52.63% to 47.33%, and the proportion of LF in plant-based foods decreased from 67.71% to 65.31%. During 2000–2011, the proportion of WF, CF, NF, EF and LF in animal-derived foods increased from 73.13% to 75.04%, 79.09–81.36%, 71.11–74.14%, 47.37–52.67% and 32.29–34.69%. 

This study also considered the differences among 12 provinces during the year of 2011. Whereas the WF of Henan households was observed to be the lowest (2.97 $m^{3}/\mathrm{cap}/d$), Guizhou was the highest (5.33 $m^{3}/\mathrm{cap}/d$), 1.79-fold higher than Henan. The EF of Chongqing household was recorded as the lowest (6.83 MJ/cap/d), while Guizhou was found highest (11.69 MJ/cap/d), 1.71-fold higher than Chongqing. Furthermore, the dietary CF and NF of Beijing households was the lowest, reached 1.85 ${\mathrm{kgCO}}_{2}\mathrm{eq}/\mathrm{cap}/d$ and 4.35 ${\times 10}^{-2}\;g/\mathrm{cap}/d$. However, the CF of Guizhou was the highest (3.19 $\;{\mathrm{kgCO}}_{2}\mathrm{eq}/\mathrm{cap}/d$), 1.72-fold higher than Beijing. The NF of Guizhou household was the highest (7.90 ${\times 10}^{-2}\;g/\mathrm{cap}/d$), 1.82-fold higher than Beijing. The LF of Shanghai household was the lowest (5.78 ${\times \;10}^{-4}\;{\mathrm{ghm}}^{2}/\mathrm{cap}/d$), while Guizhou reached the highest (1.30 ${\times \;10}^{-3}\;{\mathrm{ghm}}^{2}/\mathrm{cap}/d$), 2.24-fold higher than Shanghai. More spatial-temporal analysis and composition characteristics for multiple footprints of 12 provinces were shown in Figures S1–S6 in Supplementary Materials.

### 3.2. Spatial-Temporal Analysis for Multiple Footprints Intensity of Energy Intake

**Figure 4:**
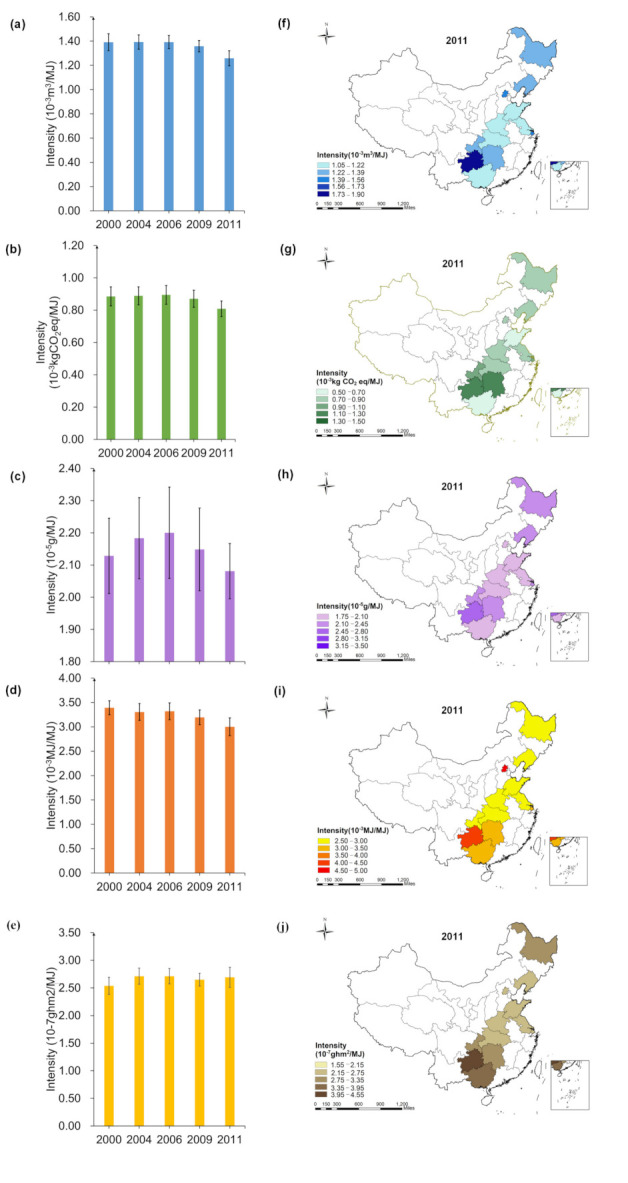
Spatial-temporal characteristics of multiple footprint intensity of energy intake ((**a**,**f**): WF; (**b**,**g**): CF; (**c**,**h**): NF; (**d**,**i**): EF; (**e**,**j**): LF; The error lines in the figure are standard errors).

As can be seen in Figure 4, ${\mathrm{EFI}}_{e}$ changed the most from 2000 to 2011, a decrease of 11.52%, while ${\mathrm{LFI}}_{e}$ increased by 6.12%.

Dietary ${\mathrm{WFI}}_{e}$, $NFI_{e}$, ${\mathrm{LFI}}_{e}$ of Guizhou household is the highest, respectively, reached 1.86 ${\times 10}^{-3}\;m^{3}/\mathrm{MJ}$, 2.76 ${\times 10}^{-5}\;g/\mathrm{MJ}$, 4.53 ${\times \;10}^{-7}{\mathrm{ghm}}^{2}/\mathrm{MJ}$. ${\mathrm{CFI}}_{e}$ of Hunan household is the highest (1.13${\times 10}^{-3}{\;\mathrm{kgCO}}_{2}\mathrm{eq}/\mathrm{MJ}$). ${\mathrm{EFI}}_{e}$ of Shandong household is the lowest (2.51 ${\times 10}^{-3}\;\mathrm{MJ}/\mathrm{MJ}$), and that of Beijing household is the highest (4.55 ${\times 10}^{-3}\;\mathrm{MJ}/\mathrm{MJ}$). 

### 3.3. Spatial-Temporal Analysis for Multiple Footprints Intensity of Protein Intake

**Figure 5:**
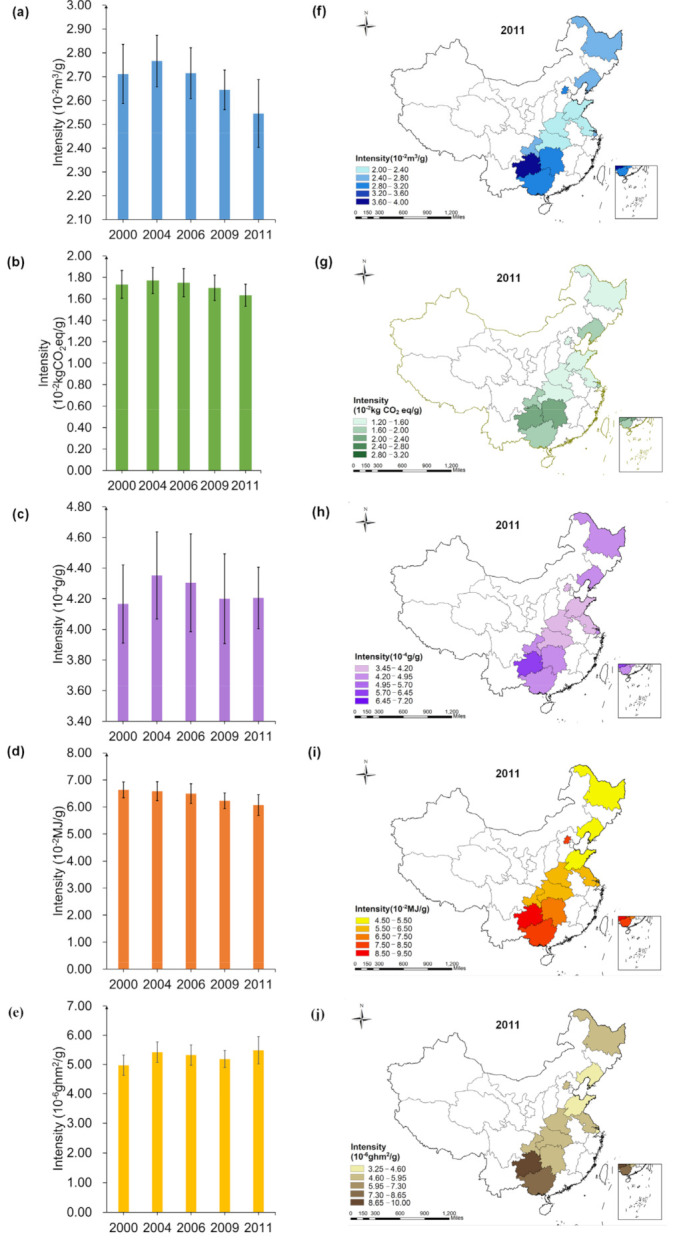
Spatial-temporal characteristics of multiple footprint intensity of protein intake ((**a**,**f**): WF; (**b**,**g**): CF; (**c**,**h**): NF; (**d**,**i**): EF; (**e**,**j**): LF; The error lines in the figure are standard errors).

In Figure 5, ${\mathrm{LFI}}_{p}$ changed the most during 2000–2011, increased by 10.23% in 2011, ${\mathrm{WFI}}_{p}$, ${\mathrm{CFI}}_{p}$ and ${\mathrm{EFI}}_{p}$ decreased by 6.12%, 5.83% and 8.46%, respectively. 

The multiple footprints intensity of nutrient intake of household in Guizhou was the highest in 2011, reached 3.99${\times 10}^{-2}\;m^{3}{/g(\mathrm{WFI}}_{p}$), 2.36 ${\times \;10}^{-2}{\;\mathrm{kg}\;\mathrm{CO}}_{2}{\mathrm{eq}/g(\mathrm{CFI}}_{p}$), 5.91 ${\times \;10}^{-4}{g/g(\mathrm{NFI}}_{p}$), 8.74 ${\times \;10}^{-2}{\;\mathrm{MJ}/g(\mathrm{EFI}}_{p}$) and 9.68 ${\times \;10}^{-6}{\;\mathrm{ghm}}^{2}{/g(\mathrm{LFI}}_{p}$), respectively. The ${\mathrm{WFI}}_{p}$, ${\mathrm{CFI}}_{p}$, ${\mathrm{EFI}}_{p}\;$and ${\mathrm{LFI}}_{p}$ of households in Shandong were the lowest, which was 2.12 ${\times \;10}^{-2}{\;m}^{3}/g$, 1.307 ${\times \;10}^{-2\;}{\mathrm{kg}\;\mathrm{CO}}_{2}\mathrm{eq}/g$, 4.82 ${\times \;10}^{-2}\;\mathrm{MJ}/g$ and 4.22 ${\times \;10}^{-6}{\mathrm{ghm}}^{2}/g,\;\mathrm{respectively}$. The ${\mathrm{NFI}}_{p}$ of household in Beijing was the lowest, which was 3.50 ${\times \;10}^{-4}\;g/g$.

### 3.4. Spatial-Temporal Analysis for Multiple Footprints Intensity of Insoluble Dietary Fiber Intake

**Figure 6:**
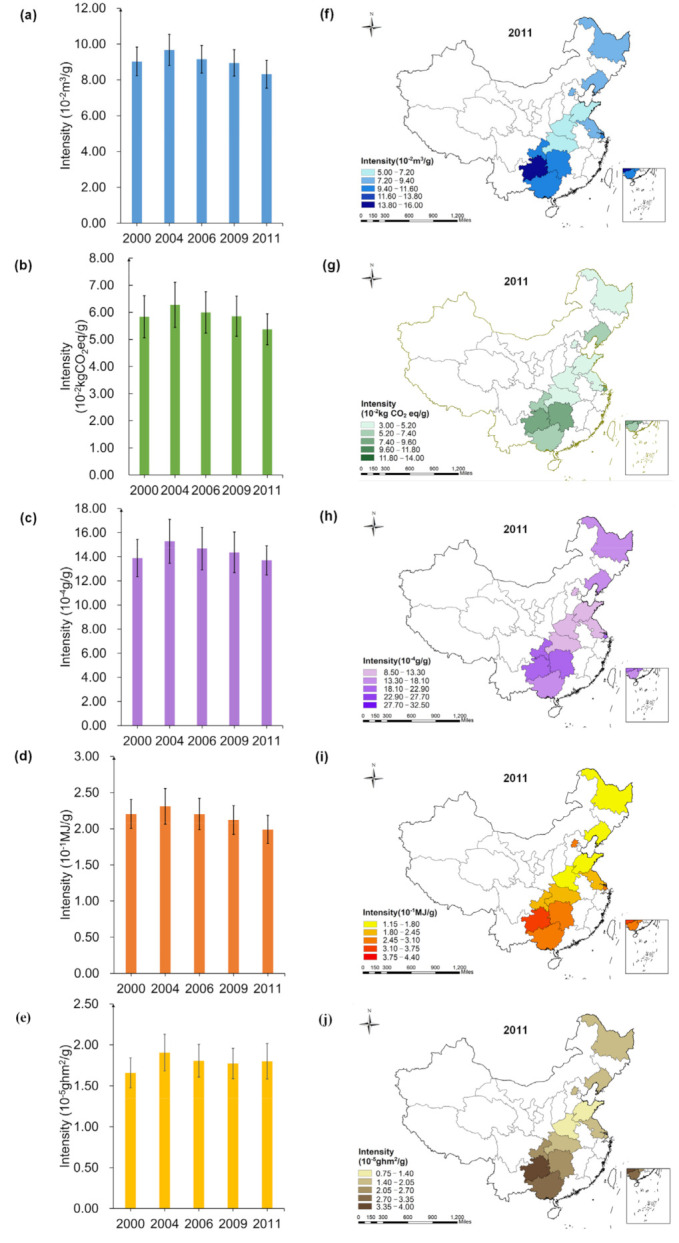
Spatial-temporal characteristics of multiple footprint intensity of insoluble dietary fiber intake (**a**,**f**): WF; (**b**,**g**): CF; (**c**,**h**): NF; (**d**,**i**): EF; (**e**,**j**): LF; The error lines in the figure are standard errors).

In Figure 6, ${\mathrm{EFI}}_{f}$ changed the most from 2000 to 2011, decreasing by 9.74%; ${\mathrm{LFI}}_{f}$ increased by 8.46%; ${\mathrm{WFI}}_{f}$, ${\mathrm{CFI}}_{f}$ and ${\mathrm{NFI}}_{f}$ decreased by 7.89%, 7.94%, 1.37%, respectively.

The multiple footprint intensity of insoluble fiber intake of household in Shandong in 2011 was the lowest, which was 5.21 ${\times \;10}^{-2}\;m^{3}/g$ (${\mathrm{WFI}}_{f}$), 3.21 ${\times \;10}^{-2}\;{\mathrm{kgCO}}_{2}{\mathrm{eq}/g\;(\mathrm{CFI}}_{f}$), 8.94 ${\times \;10}^{-4}\;g/g$ (${\mathrm{NFI}}_{f}$), 1.18 ${\times \;10}^{-1}\;\mathrm{MJ}/g$ (${\mathrm{EFI}}_{f})$ and 1.04 ${\times \;10}^{-5}\;{\mathrm{ghm}}^{2}/g$ (${\mathrm{LFI}}_{f}$), respectively. The ${\mathrm{WFI}}_{f}$ (14.52 ${\times \;10}^{-2}\;m^{3}/g$), ${\mathrm{NFI}}_{f}$ (21.51 ${\times \;10}^{-4}\;g/g$), ${\mathrm{EFI}}_{f}$ (3.18 ${\times \;10}^{-1}\;\mathrm{MJ}/g$) and ${\mathrm{LFI}}_{f}$ (3.53 ${\times \;10}^{-5}\;{\mathrm{ghm}}^{2}/g$) of household in Guizhou was the highest, which was 2.79, 2.41, 2.69 and 3.41 times than that of Shandong, respectively. The ${\mathrm{CFI}}_{f}$ of household in Hunan was the highest, reached 9.16 ${\times \;10}^{-2}\;{\mathrm{kgCO}}_{2}\mathrm{eq}/g$, 2.85 times than that of Shandong.

### 3.5. Spatial-Temporal Analysis for Multiple Footprints Intensity of Cholesterol Intake

**Figure 7:**
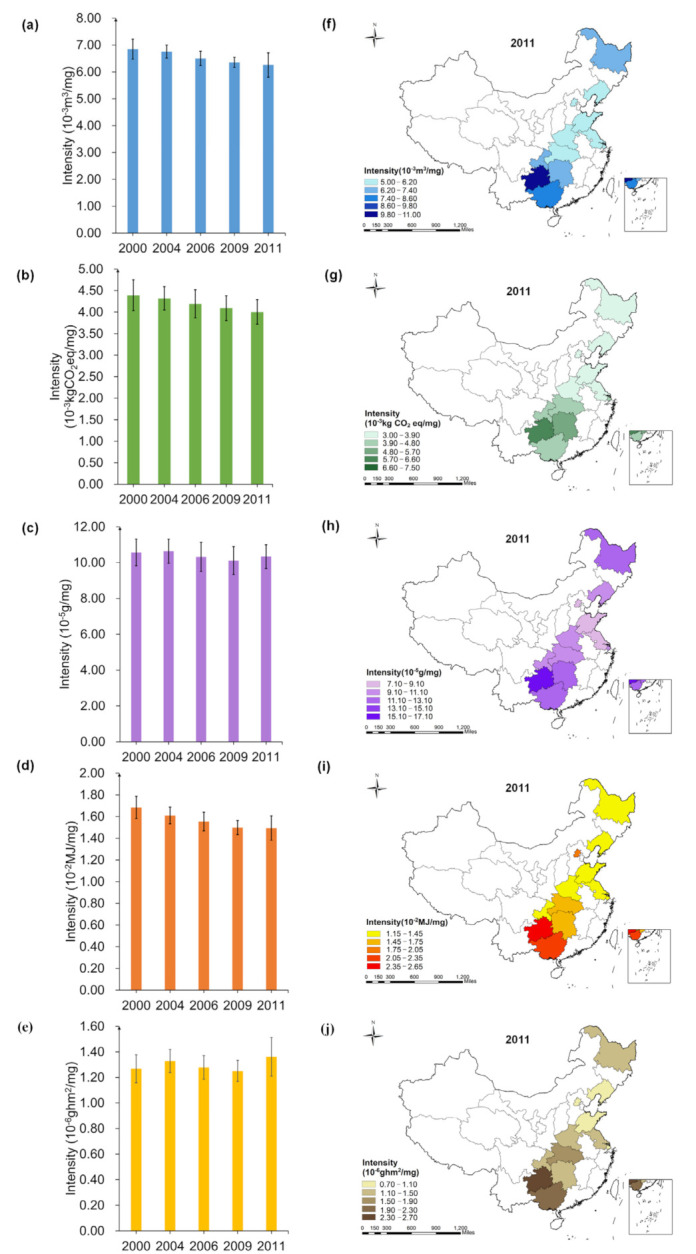
Spatial-temporal characteristics of multiple footprint intensity of cholesterol intake (**a**,**f**): WF; (**b**,**g**): CF; (**c**,**h**): NF; (**d**,**i**): EF; (**e**,**j**): LF; The error lines in the figure are standard errors).

In Figure 7, ${\mathrm{EFI}}_{c}$ changed the most during 2000–2011, decreasing by 11.31%. ${\mathrm{LFI}}_{c}$ increased by 7.34%. $\mathrm{The}\;{\mathrm{WFI}}_{c}$ $\mathrm{and}\;{\mathrm{EFI}}_{c}$ of households in Shandong in 2011 was the lowest, 5.20 ${\times \;10}^{-3}\;m^{3}/\mathrm{mg}$ and 1.18${\times 10}^{-2}\;\mathrm{MJ}/\mathrm{mg},\;\mathrm{respectively}$. ${\mathrm{The}\;\mathrm{CFI}}_{c}$ and ${\mathrm{NFI}}_{c}$ of households in Beijing was the lowest, which was 3.10 ${\times \;10}^{-3}{\;\mathrm{kgCO}}_{2}\mathrm{eq}/\mathrm{mg}$, 7.31 ${\times \;10}^{-5}\;g/\mathrm{mg},\;\mathrm{respectively}$. The ${\mathrm{LFI}}_{c}$ of households in Shanghai was the lowest (0.93${\times 10}^{-6}{\;\mathrm{ghm}}^{2}/\mathrm{mg}$).

The multiple footprint intensity of cholesterol intake of household in Guizhou was the highest, which was 10.88${\times 10}^{-3}{\;m}^{3}/\mathrm{mg}$ (${\mathrm{WFI}}_{c}$), 6.51${\times 10}^{-3}{\mathrm{kgCO}}_{2}\mathrm{eq}/\mathrm{mg}$ (${\mathrm{CFI}}_{c}$), 16.12 ${\times \;10}^{-5}\;g/\mathrm{mg}$ (${\mathrm{NFI}}_{c}$), 2.39${\times 10}^{-2}\;\mathrm{MJ}/\mathrm{mg}$ (${\mathrm{EFI}}_{c}$) and 2.64 ${\times \;10}^{-6}{\;\mathrm{ghm}}^{2}/\mathrm{mg}$ (${\mathrm{LFI}}_{c}$), respectively.

### 3.6. Driving Factors of Multiple Footprints of Food Consumption

**Figure 8:**
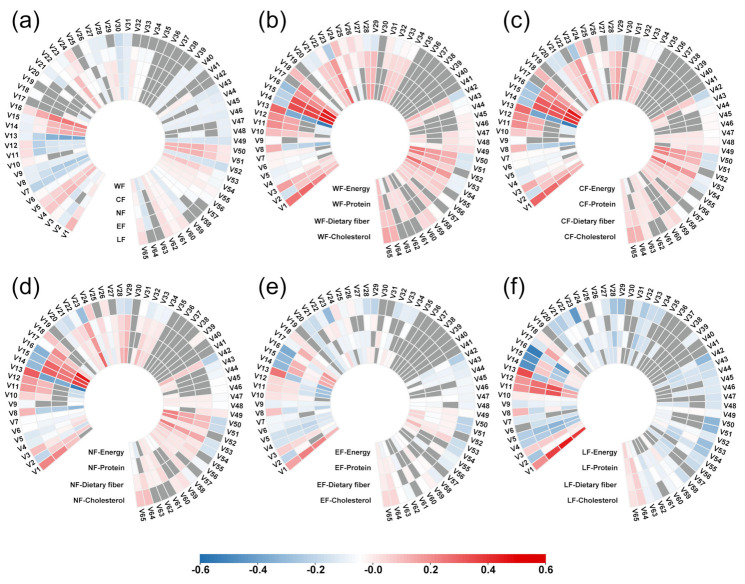
Correlation between multiple footprints and driving factors (Note: (**a**) Multiple footprint; (**b**) WFI; (**c**) CFI; (**d**) NFI; (**e**) EFI; (**f**) LFI).

In Figure 8, most of the proposed independent variables showed different degrees of correlation with multiple footprints and multiple footprints intensity of nutrient intake. The nutrient intake illustrated a strong correlation with multiple footprint and nutrient intake intensity. In Figure 8a, among all the correlations, the proportion of protein intake (V16) and WF presented the strongest positive correlation, while the proportion of carbohydrate intake (V13) and WF had the strongest negative correlation. It can be found that the driving factors of WF, CF and NF are consistent to some extent. Among the 65 proposed independent variables, 53 (81.54%) were significantly correlated with the WF, while 46 (70.77%) correlated with the LF. In Figure 8b, it illustrated that the strongest positive correlation was found between proportion of fat intake (V14) and ${\mathrm{WFI}}_{e}$, and a strong negative correlation was found among proportion of carbohydrate intake (V13) and ${\mathrm{WFI}}_{e}$. A certain degree of consistency was observed among the driving factors of ${\mathrm{WFI}}_{e}$, ${\mathrm{WFI}}_{p}$ and ${\mathrm{WFI}}_{f}$, where 49 (75.38%) of the variables correlated with ${\mathrm{WFI}}_{c}$, and 43 (66.15%) correlated with ${\mathrm{WFI}}_{p}$.

Figure 8c shows that the strongest positive correlation was found among the proportion of protein intake (V16) and ${\mathrm{CFI}}_{e}$, and negative correlation was obtained among carbohydrate intake (V13) and ${\mathrm{CFI}}_{e}$. Among these, 49 (75.38%) correlated with ${\mathrm{CFI}}_{f}$, and 43 (66.15%) negatively correlated with ${\mathrm{CFI}}_{p}$. Figure 8d shows a strong positive correlation between protein intake (V16) and ${\mathrm{NFI}}_{e}$, while there was a negative correlation between carbohydrate intake (V13) and ${\mathrm{NFI}}_{e}$. It can be seen that 53 (81.54%) variables correlated with the ${\mathrm{NFI}}_{e}$, and 42 (64.62%) correlated with ${\mathrm{NFI}}_{p}$. According to Figure 8e, a strong positive correlation was observed between proportion of carbohydrate intake (V13) and ${\mathrm{EFI}}_{c}$, the strongest negative correlation was observed between protein intake (V16) and ${\mathrm{EFI}}_{c}$. Overall, 52 (80.00%) variables correlated with ${\mathrm{EFI}}_{c}$ and 43 (66.15%) correlated with ${\mathrm{EFI}}_{p}$. In Figure 8f, among all the correlations, the strongest correlation was province (V1) and ${\mathrm{LFI}}_{p}$. There were 53 (81.54%) proposed independent variables that correlated with ${\mathrm{LFI}}_{c}$, and 40 (61.54%) correlated with ${\mathrm{LFI}}_{e}$.

### 3.7. Scenario Analysis of Different Dietary Patterns

This study selected China’s food consumption data during 2011 as the basic scenario (baseline). These results were compared with two developed countries: the United States (S1) and Japan (S2). The United States was selected because it is the most developed country [41], while Japan was selected because it borders China and has a similar household dietary habit to China [42]. Additionally, more reliable data from 2015 can be obtained for the United States and Japan. The study also chose Chinese Dietary Guidelines 2016 (S3) [43], recommended food consumption of Guideline of Australia 2013 (S4) [44], and the food consumption guidelines issued by Germany 2013 (S5) as reference scenarios [45].

**Figure 9:**
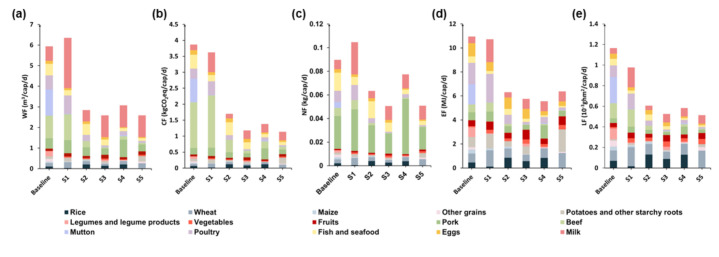
Scenarios analysis for multiple footprints of household under different dietary patterns ((**a**) WF; (**b**) CF; (**c**) NF; (**d**) EF; (**e**) LF; Baseline: CHN 2011 [37]; S1: US 2015 [41]; S2: JPN 2015 [42]; S3: Chinese Dietary Guidelines 2016 [43]; S4: Guideline of Australia 2013 [44]; S5: guidelines issued by Germany 2013 [45]).

The results from Figure 9 demonstrated that the CF, EF and LF of China’s food consumption pattern during 2011 were highest among all scenarios. The WF and NF dietary pattern recommended by China were found to be the lowest among several scenarios. When the dietary pattern reaches the recommended value of Chinese Dietary Guidelines 2016, the WF, CF, NF, EF and LF would be reduced by 56.48%, 69.47%, 43.57%, 47.44%, and 54.91%, respectively. The WF, NF and LF of plant-foods consumed by Chinese households in 2011 were the highest among all scenarios, and the CF, EF and LF of animal-derived foods consumed by Chinese households in 2011 were also the highest among all scenarios. Meanwhile, the biggest reduction in the consumption of plant-based foods would be the WF, which would reduce by 31.14%, and the biggest reduction in the consumption of animal-derived foods would be the CF, which would reduce by 75.53%.

**Figure 10:**
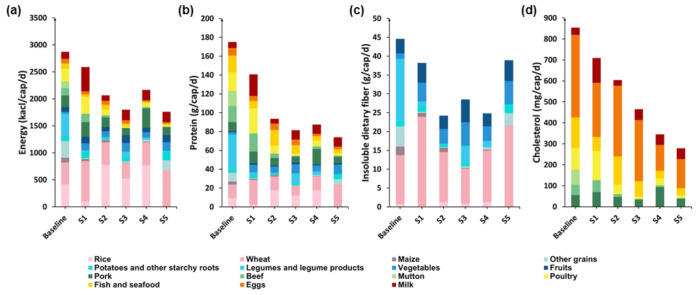
Scenarios analysis for nutrient intake of household under different dietary patterns ((**a**) energy intake; (**b**) protein intake; (**c**) insoluble dietary fiber intake; (**d**) cholesterol intake; Baseline: CHN 2011 [37]; S1: US 2015 [41]; S2: JPN 2015 [42]; S3: Chinese Dietary Guidelines 2016 [43]; S4: Guideline of Australia 2013 [44]; S5: guidelines issued by Germany 2013 [45]).

Due to the different dietary habits, there were significant differences in nutrient intake among households in different countries. As can be seen from Figure 10, the nutrient intakes of Chinese households in 2011 are the highest, which were higher than the intakes recommended in the Chinese Dietary Guidelines 2016. The energy intake of Chinese households in 2011 was the highest (2870.17 kcal/cap/d), and the food consumption guidelines issued by Germany 2013 were the lowest (1762.66 kcal/cap/d). In 2015, American household consumption of animal-derived foods provided the highest energy (1849.87 kcal/cap/d), while German dietary guidelines recommended that animal-derived foods provided the lowest energy (432.99 kcal/cap/d). In 2011, Chinese household food consumption of plant-based foods provided the highest protein (80.40 g/cap/d), while that of Japan (2015) was the lowest (43.68 g/cap/d). In 2011, China’s household food consumption of animal-derived foods provided the highest protein (94.79 g/cap/d), and that of animal-derived foods recommended by German dietary guidelines provided the lowest protein (27.19 g/cap/d). In 2011, the dietary protein intake of Chinese households was higher than that of the United States and Japan, but the intake of high-quality protein from aquatic products and other animal-derived foods was lower. However, the intake of nutrients under the recommended recipes in Australia and the dietary guidelines in Germany was relatively low, and the footprint value was also relatively low. The intake of nutrients was in line with the dietary pattern recommended by the Chinese Dietary Guidelines 2016, which could be used as a reference.

## 4. Discussion

### 4.1. Driving Mechanism of Household Dietary Footprints

Results show that multiple footprints of household food consumption have strong correlation with household nutrient intake. The water footprint of animal-derived foods is higher than plant-based foods, consequently presenting greater impacts, particularly on water footprint [29]. It can be seen that household animal-derived protein and cholesterol intakes increased with the proportions of animal-derived foods in household dietary pattern. This trend might increase the pressure on shortages of water resources [46]. From 2000 to 2011, the dietary energy of urban and rural residents in China mainly came from cereals (or carbohydrates); the protein mainly came from cereals [47]. Additionally, the results showed that the proportion of carbohydrate intake was negatively correlated to multiple footprints. To a certain extent, it indicates that a dietary pattern partial to carbohydrate intake is more environmentally friendly than a dietary pattern partial to high animal protein and cholesterol intake. Previous research demonstrated that each person should reduce their consumption of animal-derived foods by 205.1 kg ${\mathrm{CO}}_{2},$ equivalent to 12.1% per year [48]. Other studies reported that, in order to support health and achieve the climate stability goal, beef could be replaced with pea protein to reduce the environmental footprint of animal-derived foods [49,50]. Greenhouse gas (GHG) emitted from the production of plant-based beverages (such as oats, soybeans, almonds and rice milk) contributes only 22–38% to the total greenhouse gas emitted from the milk production. Thus, replacing milk and other dairy products with plant-based beverages could also greatly reduce water consumption [51].

Footprints of household food consumption shows a strong correlation with educational levels. The proportion of carbohydrate, protein and fat intake had changed with the change of household dietary pattern in China, which led to a great impact on water and carbon footprint. As shown in the study of high carbohydrate intake among adult women in Botswana, educational level may influence the choice of food intake [52]. The increase in education level would directly increase the consumer’s attention to the rationality of nutrient intake, and tended to increase the intake of protein instead of calories [53]. The educational level also affects the household choice of proportion of animal-derived and plant-based foods [54], which directly affects household intake of carbohydrate, protein and energy, and indirectly drives the change of multiple footprints of household food consumption.

With the increase in income status, people tended to increase nutrient intake. However, people with a higher income status tends to increase the consumption of more refined foods, price level and taste grade [55]. The increasing complexity of food processing also increased the multiple footprints of food. Due to different income status of household in different provinces, or urban and rural areas, variations among household eating habits are formed. A previous study argued that the more developed and urbanized a household, the more likely it was to have a higher sugar, fat, and highly processed and packaged food intake [56]. Hence, rising income and urbanization effectively drives the dietary transition, where the traditional diets are replaced by diets with more refined sugars, refined fats, oils and meats [57]. Households of developed provinces increase the consumption of various animal-derived foods (particularly poultry and pork), while in rural areas, households increase the consumption of pork. Study shown the urban and rural households have no strong preference to beef and mutton. Rural households who increase the consumption of poultry, beef and mutton will reduce their consumption of pork and increase consumption of other animal-derived foods [58]. These results illustrated the differences of household food consumption footprints between urban and rural areas. Therefore, provinces and urbanization are the key factors for driving of household food consumption footprints.

The convenience of public and private transportation reduced the calorie consumption of households, and high-fat and high-protein foods become cheaper. Urbanization promoted household incomes together with the increasing consumption of such foods. People can get more expensive calories from non-starch foods, so they choose to reduce the consumption of rice and flour [59]. Currently, China is experiencing the transition from a developing to a developed country, having the influence of huge population and trend of developing economic, household food waste became serious issue, resulting in the unnecessary consumption of resources [60].

Among 12 provinces involved in the study, the results have shown that the multiple footprints of food consumption were highest for households in Guizhou. Guizhou is rich in natural products and diverse in diet. The terrain in Guizhou is complex and mainly mountainous. The mild and humid climate also brings Guizhou an advanced planting industry [61]. As shown in our study, the beef consumption of household in Guizhou (35.57 g/cap/d) was not higher than most provinces. However, the multiple footprints of unit yield of beef in Guizhou were the highest among the 12 provinces. Thus, the WF (0.66 $m^{3}/\mathrm{cap}/d$), CF (1.33 ${\mathrm{kgCO}}_{2}\mathrm{eq}/\mathrm{cap}/d$), NF (3.20 ${\times 10}^{-2}\;g/\mathrm{cap}/d$) and LF (0.20 ${\times \;10}^{-4}{\;\mathrm{ghm}}^{2}/\mathrm{cap}/d$) of beef consumption of household in Guizhou is the highest among 12 provinces, while the EF (0.33 MJ/cap/d) is also higher than most of the provinces. This situation also appears in several other animal-derived foods. Moreover, because the multiple footprints of per unit yield of animal-derived foods are higher than that of plant-based foods, the dietary multiple footprints of households in Guizhou are higher than that of other provinces. In order to achieve sustainable development, it is suggested that Guizhou should reduce their consumption of animal-derived foods by utilizing the unique Karst landform and the potential of the grassland to develop its animal husbandry [62].

### 4.2. Suggestions for Sustainable Dietary Adjustment

Based on the above, the dietary pattern of households in China is not a sustainable form of development; the following suggestions are proposed: (i) we suggest that households should reduce the consumption of red meat (mutton, beef, etc.), because the multiple footprints of unit yield for red meat is higher than other kinds of foods (e.g., the WF of mutton was 14.55 $m^{3}/\mathrm{kg}$, 92.46 times higher than that of vegetables). We also suggest that households should consider conforming to a balanced diet, reducing the excessive intake of food and keeping food waste to a minimum. Based on the increasing demand for animal food, we suggest using plant-based foods rich in protein as a substitute for some animal-derived foods, and swap refined grain choices for whole grains, which not only meet household requirements for nutrient intake and health keeping, but also achieve the purpose of reducing the consumption of dietary resources [49,50,63]. Residents should be encouraged to get protein through beans, using plant-based beverages instead of milk and other animal dairy products [51]. Food waste, reduce resource consumption and greenhouse gas emissions caused by the loss of food production and consumption [64] should be reduced. We also suggest that people with higher income and educational level adjust their dietary pattern according to dietary guidelines issued by China (2016) [43], avoiding the blind pursuit of the high-sugar and high-fat dietary pattern common in Western or developed countries, reduce excessive intake of nutrients, and choose foods with the same nutritional value and lower resource footprint [53]; (ii) we also suggest that the Chinese Government encourages the development of sustainable production and processing technology in the agriculture and food processing industry, and improve household awareness of dietary guidelines. Additionally, we suggest that the Chinese Government pays more attention to guiding consumers, such as publicity and education, and spreads the use of footprint calculators or footprint price lists for households, making dietary choices clearer; (iii) we suggest that nutrient research institutions consider household nutrient intake alongside resource consumption when formulating recommended dietary pattern.

Future studies should focus on the social response to dietary multiple footprints and methods of reducing resources consumption and GHG emissions globally. Further improvements in data integrity with better classification of foods and broader survey are needed to provide a more sophisticated understanding of the influences of diet on environment and sustainable development. A more comprehensive understanding of the impact that the driving mechanism of household dietary has on the multiple footprints on scales will facilitate households, governments and research institutes to adjust dietary patterns, accelerate technological innovation and further studies on the relationship between food consumption and environmental impact, alleviating the current resource and environmental pressures to achieve absolute sustainability.

## 5. Conclusions

This paper studied the spatial-temporal characteristics of multiple dietary footprints and analyzed its driving mechanisms. The main conclusions were as follows:

(1) During 2000–2011 in China, household dietary consumption WF, CF, NF and EF were decreased by 18.24%, 17.82%, 12.03% and 20.36%, respectively. However, the LF remained stable within the time scale of the research. For footprint intensity, the ${\mathrm{EFI}}_{e}\;\left(-11.52\%\right)$, ${\mathrm{EFI}}_{f}\;$(−9.74%) and ${\mathrm{EFI}}_{c}\;$(−11.31%) have decreased, while ${\mathrm{LFI}}_{p}$, and ${\mathrm{LFI}}_{c}$ have increased by 10.23% and 7.34%, respectively.
(2) Nutritional intake ratio, household attributes, educational and health consciousness had a stronger correlation with dietary footprints. Among these, protein intake had a stronger positive correlation with WF, ${\mathrm{NFI}}_{e}$ and ${\mathrm{CFI}}_{e}$, while the proportion of carbohydrate intake had a stronger negative correlation with WF, ${\mathrm{WFI}}_{e}$, ${\mathrm{CFI}}_{e}$ and ${\mathrm{NFI}}_{e}$. The proportion of fat intake had a stronger positive correlation with ${\mathrm{WFI}}_{e}$. The proportion of carbohydrate intake had a stronger positive correlation with ${\mathrm{EFI}}_{c}$, and the proportion of protein intake that had a stronger negative correlation was ${\mathrm{EFI}}_{c}$.
(3) The multiple footprints of Chinese household food consumption are much higher than the dietary pattern recommended in the Chinese Dietary Guidelines 2016. It is strongly suggested that the households reduce animal-derived foods with high footprints (especially beef and mutton). Additionally, household should increase the intake of fruits and vegetables to reduce the size of the multiple footprints.

## Figures and Tables

**Table 1:** Food Classification.

Primary Classification	Secondary Classification	Food Type
Cereals and starchy roots	Wheat	Plant-based foods
	Maize	Plant-based foods
	Rice	Plant-based foods
	Other grains	Plant-based foods
	Potatoes and other starchy roots	Plant-based foods
Legumes and legume products	Legumes and legume products	Plant-based foods
Vegetables and fruits	Vegetables	Plant-based foods
	Fruits	Plant-based foods
Animal-derived foods	Beef	Animal-derived foods
	Pork	Animal-derived foods
	Poultry	Animal-derived foods
	Mutton	Animal-derived foods
	Fish and seafood	Animal-derived foods
	Eggs	Animal-derived foods
	Milk	Animal-derived foods

**Table 2:** List of independent variables.

Type	Sample Size	Variable	Code
Household attributes	63,550	Province	V1
	63,550	Survey year	V2
	63,550	Nationality	V3
	59,074	Height (cm)	V4
	58,895	Weight (kg)	V5
	63,550	Calculated age in years to 0 decimal points	V6
	63,550	Gender	V7
	63,032	Urban site or rural site	V8
	54,564	Marital status	V9
	3436	Is R a national minority	V10
	3413	R’s birthplace	V11
	3403	R’s “old home”(father’s birthplace)	V12
Nutritional intake ratio	63,414	3-day average: carbohydrate (g)	V13
	63,414	3-day average: fat (g)	V14
	63,414	3-day average: energy (kcal)	V15
	63,414	3-day average: protein (g)	V16
Income status	14,288	Work in HH garden/orchard last year	V17
	12,311	Number Of months farmed last year	V18
	12,647	Type of farming business	V19
	13,000	Individual farming income (Yuan)	V20
	15,071	Individual gardening income (Yuan)	V21
	8420	Individual livestock income (Yuan)	V22
	39,993	Total net individual income (Yuan)	V23
	53,168	Presently working?	V24
	32,017	Primary occupation	V25
	32,084	Has a secondary occupation	V26
	26,115	Average of days/week worked last year (Day)	V27
	25,928	Average of hours/day worked last year (h)	V28
	12,709	Average monthly wage last year (Yuan)	V29
Health and medical conditions	1242	B-feeding: ever breastfed child?	V30
	49,407	Been sick or injured in last 4 weeks?	V31
	20,269	Monthly contribution to medical insurance (Yuan)	V32
	40,943	Priorities: physically active	V33
	40,938	Priorities: healthy diet	V34
	58,834	Blindness in 1 eye?	V35
	58,834	Blindness in both eyes?	V36
	58,833	Loss of 1 arm or the use of 1 arm?	V37
	58,834	Loss of both arms or use of both arms?	V38
	58,829	Loss of 1 leg or the use of 1 leg?	V39
	58,813	Loss of both legs or use of both legs?	V40
	14,050	Currently pregnant?	V41
	62,609	Do you have medical insurance?	V42
	54,719	Diagnosed with high blood pressure?	V43
	54,422	Diagnosed with diabetes?	V44
	52,037	Diagnosed with myocardial infarction?	V45
	51,879	Diagnosed with apoplexy?	V46
	12,828	Doctor’s diagnosis of illeness/injury: tumor	V47
	54,818	History of bone fracture?	V48
	30,775	Comparative health status	V49
Educational level and social life	55,024	Years of education in regular school	V50
	59,880	Highest level of education attained	V51
	58,951	Currently in school?	V52
	45,874	Ever go to internet cafe	V53
	44,385	Know about Chinese dietary guidelines	V54
Living habits	7188	School: Do physical exercises?	V55
	7769	Body shapes: looks most like you	V56
	6736	Weight: under/normal/over	V57
	7891	On a diet last year?	V58
	7388	Physical activity: too little/right/too much	V59
	54,913	Ever smoked cigarettes?	V60
	14,083	Number of cigarettes smokes per day	V61
	54,757	Drank beer/alcohol last year?	V62
	16,935	Frequency of alcohol consumption	V63
	25,928	Memory test: rate present life	V64
	11,116	Like to eat hot pepper or spicy food?	V65

## Data Availability

The data presented in this study are available on request from the corresponding author.

## Supplementary Materials

The following are available online at https://www.mdpi.com/article/10.3390/foods10081858/s1, Figure S1: Spatial-temporal analysis and composition characteristics for multiple footprints of 12 provinces in 2000, 2004, 2006, 2009, 2011, Figure S2: Spatial-temporal analysis for WF, Figure S3: Spatial-temporal analysis for CF, Figure S4: Spatial-temporal analysis for NF, Figure S5: Spa-tial-temporal analysis for EF, Figure S6: Spatial-temporal analysis for LF.
