# Supplementary material for: Spatial-Temporal Footprints Assessment and Driving Mechanism of China Household Diet Based on CHNS
Source: Foods. 2021 Aug 11;10(8):1858. doi: 10.3390/foods10081858 (PMC8391238; doi:10.3390/foods10081858)
Supplement: Supplementary file 1 [file foods-10-01858-s001.zip › foods-1288035-supplementary.pdf]

Supplementary Materials for:

Spatial-temporal footprints assessment and driving mechanism of China household diet based on CHNS

Yanling Long [1,2], Runzhi Hu [1,2], Tuo Yin [1,2], Pengxiang Wang [1,2], Jiamin Liu [1,2], Tahir Muhammad [1,2], Xiuzhi Chen [1,2,*], Yunkai Li [1,2]

[1] College of Water Resources and Civil Engineering, China Agricultural University, Beijing 100083, China.
[2] Engineering Research Center for Agricultural Water-Saving and Water Resources, Ministry of Education, Beijing 100083, China.
* Correspondence: chxz@cau.edu.cn; Tel.: 86-106-2738-485

This supplementary file includes:

Section 1. Spatial-temporal analysis and composition characteristics for multiple footprints of food consumption

Figure S1. Spatial-temporal analysis and composition characteristics for multiple footprints of 12 provinces in 2000, 2004, 2006, 2009, 2011

Section 2. Spatial-temporal analysis for multiple footprints of food consumption

Figure S2. Spatial-temporal analysis for WF

Figure S3. Spatial-temporal analysis for CF

Figure S4. Spatial-temporal analysis for NF

Figure S5. Spatial-temporal analysis for EF

Figure S6. Spatial-temporal analysis for LF

Section 1. Spatial-temporal analysis and composition characteristics for multiple footprints of food consumption

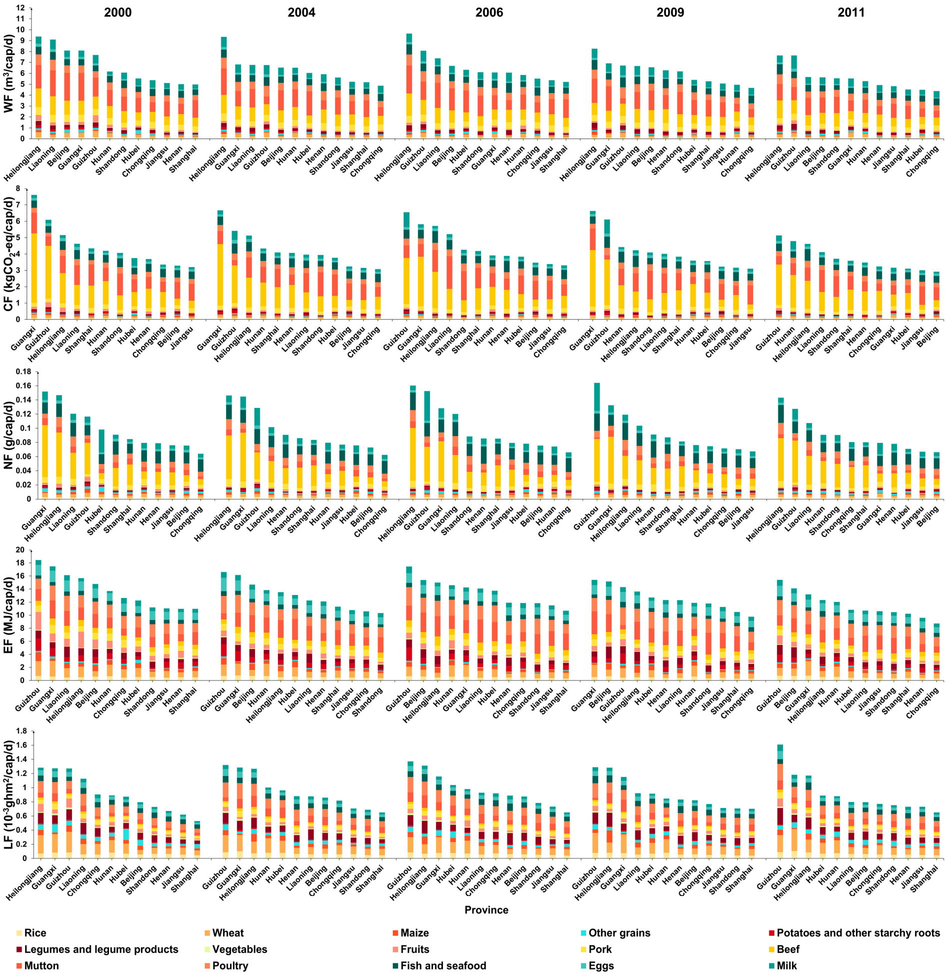

Figure S1. Spatial-temporal analysis and composition characteristics for multiple footprints of 12 provinces in 2000, 2004, 2006, 2009, 2011 (a: WF; b: CF; c: NF; d: EF; e: LF).

As shown in Figure S1, in 2000, the highest food consumption WF was 9.38 m^3/cap/d, in Heilongjiang, while the lowest was 4.98 m^3/cap/d, in Shanghai. In 2011, the highest food consumption WF was 7.64 m^3/cap/d, in Heilongjiang, while the lowest was 4.38 m^3/cap/d, in Chongqing.

In 2000, the highest food consumption CF was 7.61 $kgCO_2eq$/cap/d, in Guangxi, while the lowest was 3.20 $kgCO_2eq$/cap/d , in Jiangsu. In 2011, the highest food consumption CF was 5.14 $kgCO_2eq$/cap/d, in Guizhou, while the lowest was 2.93 $kgCO_2eq$/cap/d, in Beijing.

In 2000, the highest food consumption NF was 0.15 g/cap/d, in Guangxi, while the lowest was 0.06 g/cap/d, in Chongqing. In 2011, the highest food consumption NF was 0.14 g/cap/d, in Heilongjiang, while the lowest was 0.07 g/cap/d, in Beijing.

In 2000, the highest food consumption EF was 18.45 MJ/cap/d, in Guizhou, while the lowest was 10.97 MJ/cap/d, in Chongqing. In 2011, the highest food consumption EF was 15.41 MJ/cap/d, in Guizhou, while the lowest was 8.74 MJ/cap/d, in Chongqing.

In 2000, the highest food consumption LF was 1.28 $\times10^{-3}ghm^2$/cap/d, in Heilongjiang, while the lowest was 0.53 $\times10^{-3}ghm^2$/cap/d, in Chongqing. In 2011, the highest food consumption LF was 1.61$\times10^{-3}ghm^2$/cap/d, in Guizhou, while the lowest was 0.65 $\times10^{-3}ghm^2$/cap/d, in Shanghai.

Section 2. Spatial-temporal analysis for multiple footprints of food consumption

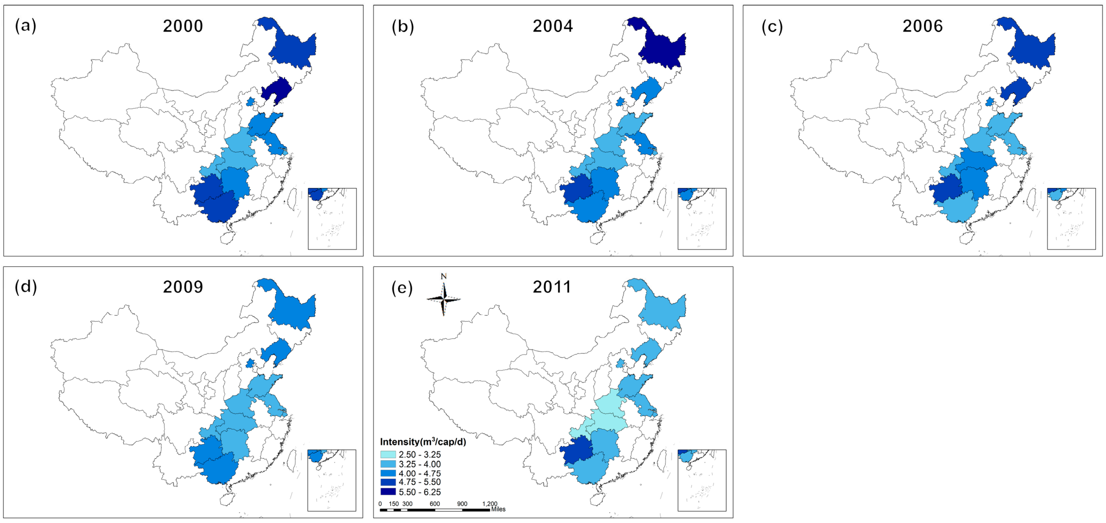

Figure S2. Spatial-temporal analysis for WF (a: year of 2000; b: year of 2004; c: year of 2006; d: year of 2009; e: year of 2011).

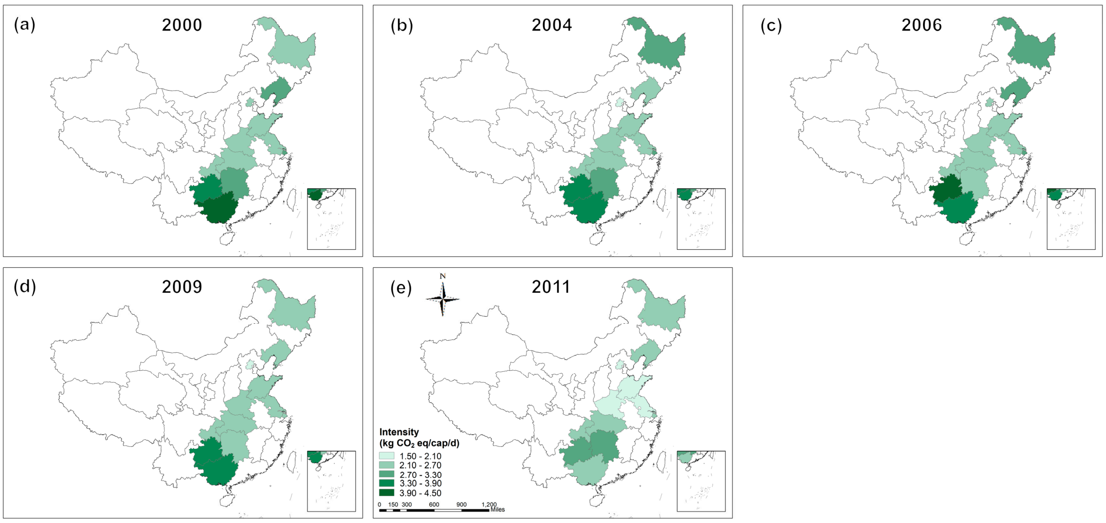

Figure S3. Spatial-temporal analysis for CF (a: year of 2000; b: year of 2004; c: year of 2006; d: year of 2009; e: year of 2011)

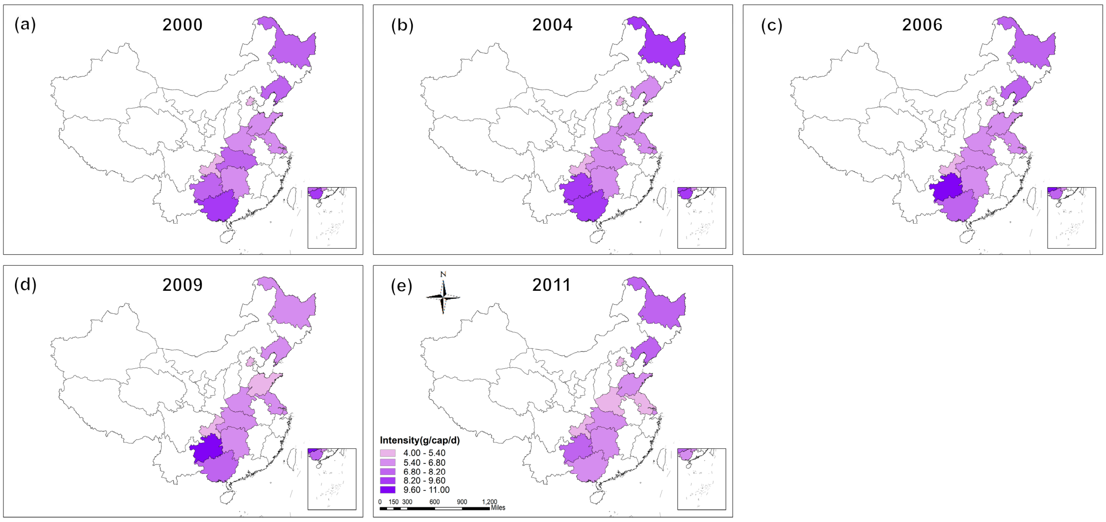

Figure S4. Spatial-temporal analysis for NF (a: year of 2000; b: year of 2004; c: year of 2006; d: year of 2009; e: year of 2011)

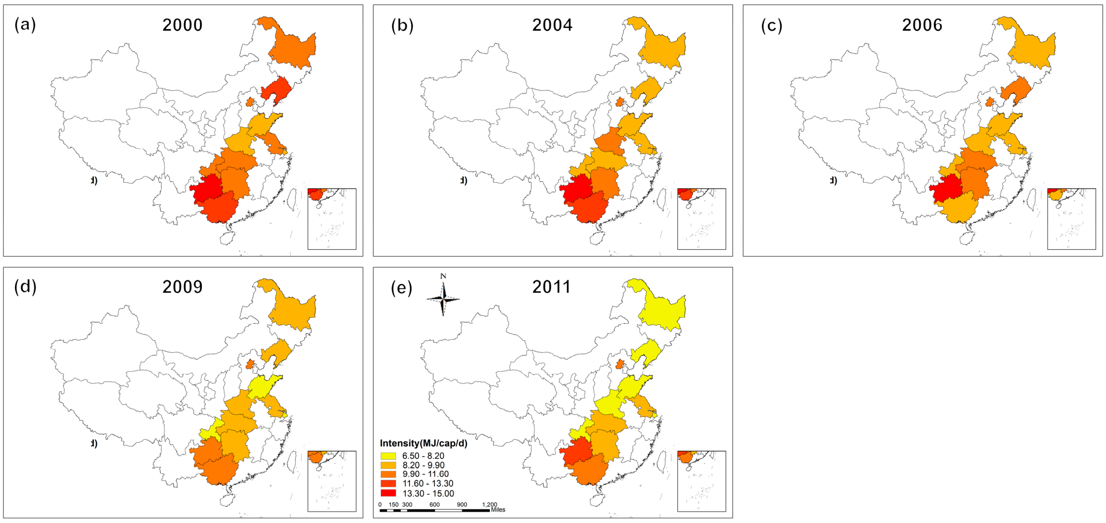

Figure S5. Spatial-temporal analysis for EF (a: year of 2000; b: year of 2004; c: year of 2006; d: year of 2009; e: year of 2011)

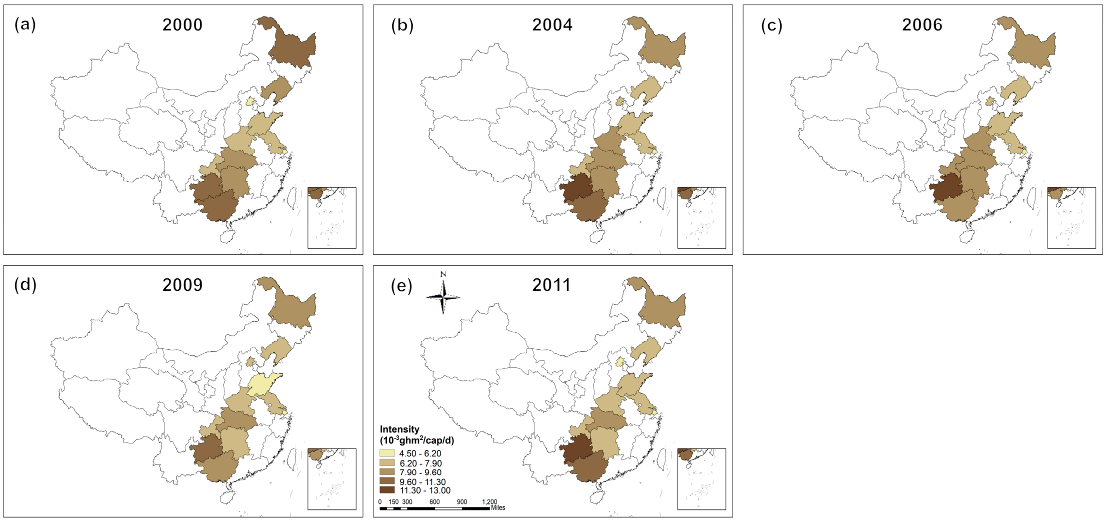

Figure S6. Spatial-temporal analysis for LF (a: year of 2000; b: year of 2004; c: year of 2006; d: year of 2009; e: year of 2011)
